# A Visual Tracking System for Honey Bee (Hymenoptera: Apidae) 3D Flight Trajectory Reconstruction and Analysis

**DOI:** 10.1093/jisesa/ieab023

**Published:** 2021-04-16

**Authors:** Cong Sun, Patrick Gaydecki

**Affiliations:** School of Electrical and Electronic Engineering, University of Manchester, Manchester M13 9PL, UK

**Keywords:** insect behavior analysis, 3D visual tracking, image processing

## Abstract

We describe the development, field testing, and results from an automated 3D insect flight detection and tracking system for honey bees (*Apis mellifera* L.) (Hymenoptera: Apidae) that is capable of providing remarkable insights into airborne behavior. It comprises two orthogonally mounted video cameras with an observing volume of over 200 m^3^ and an offline analysis software system that outputs 3D space trajectories and inflight statistics of the target honey bees. The imaging devices require no human intervention once set up and are waterproof, providing high resolution and framerate videos. The software module uses several forms of modern image processing techniques with GPU-enabled acceleration to remove both stationary and moving artifact while preserving flight track information. The analysis system has thus far provided information not only on flight statistics (such as speeds and accelerations), but also on subtleties associated with flight behavior by generating heat maps of density and classifying flight patterns according to patrol and foraging behavior. Although the results presented here focus on behavior in the locale of a beehive, the system could be adapted to study a wide range of airborne insect activity.

Among insect species, bees (Hymenoptera: Apidae) are the most important pollinators for plants and flowering crops, both in the natural and managed environment, contributing to 35% of crop production and 87% of the leading food crops across the whole world (Klein et al. 2006). However, the inflight behavior of bees has not been investigated systematically and is still a relatively unworked area. As has been frequently documented, the population of bees has been declining dramatically in recent years, particularly in North America and Europe (Reyes Tirado 2013, Potts et al. 2010). Central Europe has experienced a 25% decline in honey bee colonies, with a 54% loss in the United Kingdom since 1985 (Potts et al. 2010). These numbers are very concerning, but worse, do not even consider the decline in numbers of native wild bees (Abou-Shaara 2014). Habitat loss caused by urbanization, the use of pesticides, and infection from diseases and parasites are the main reasons often cited (Oldroyd 2007, Brown and Paxton 2009, Grossman 2013). Considering the urgent need for preventing further population decline, systematic studies of bee behavior are of considerable significance. The purpose of such studies is not initially intervention, but observation, using suitable imaging and tracking techniques. The knowledge acquired will assist with pollination management and the mitigation of population reduction; further, the instrumentation will lend itself to the study of both beneficial and pestiferous insect species. The flight behavior of bees varies across bee species because they have different morphologies (Suwannapong et al. 2012). There are also many factors that affect bee foraging preferences, including the availability of suitable plant resources (Sushil et al. 2013), ambient temperature (Tan et al. 2012), natural predators and parasites, use of insecticides, presence of diseases (Abou-Shaara 2014), the time of day, and season. Trajectory analysis will enable correlates to be established between these parameters and behavior at a detailed, quantitative level.

Research on insect observation and tracking has traditionally relied on two approaches—radar and video cameras. Vertical-looking radars can perform automatic, long-distance monitoring of flying insects over a wide altitude range (Chapman et al. 2002). These radars are relatively inexpensive and work during night hours but have dead zones (low altitude and close range) and only detect large swarms of insects during migration. Harmonic radars are a perfect solution for low altitude, single target tracking in a highly cluttered environment (Lövei et al. 1997, Capaldi et al. 2000, Fry et al. 2000, Svensson et al. 2001) but require transponders to be attached to the insects. Although small, they represent a significant fraction of the weight of the insect; further, they can disrupt the aerodynamics of the insect, thereby affecting behavior and yielding unrepresentative data. Additionally, the returned signals from the transponders contain no identification information, limiting the application in multiple target tracking.

However, the term *relatively inexpensive* is itself a qualified term; the average component cost of a vertical-looking radar is around $30,000 (Chapman et al. 2003), which is certainly beyond the research budget of most field entomologists. Tracking using video cameras, a methodology that certainly merits the accolade of *low-cost* and in the case of our system—less than £3000, has mostly been confined to laboratory studies conducted under artificial illumination using light traps or insects constrained in transparent tubes or chambers (Fry et al. 2000, Bergou et al. 2010, Spitzen et al. 2013). Modern image processing techniques based on machine learning and neural networks are capable of detecting and tracking small objects with exquisite precision; such objects may include distant ships (Bertinetto et al. 2016), pedestrians and crates on the street (He et al. 2020). The development of computer vision techniques raised the interest among entomologists in the application of insect tracking based on video, but early progress was limited. Related works have mostly been confined to 2D tracking (Balch et al. 2001, Campbell et al. 2008), and very few involved 3D (Chiron et al. 2013). The target distances were very short (1–2 m) since the relative size of the target shrinks as the observation volume expands, affecting the visibility and thereby the accuracy; this is particularly important in the tracking of small and fast-moving insects. The relatively small detection range and low reliability in highly cluttered environments limited the general application of existing systems. Therefore, the object of this publication is to seek more possibilities in understanding insect flight behaviors with modern image processing techniques with much larger observation volumes and better performance with cluttered backgrounds. The concept behind this project dates back to a system that was developed in 1982, observing nocturnal moth flight behavior in the vicinity of a mercury vapor light source (Gaydecki 2019). Although the research was successful, the data volume and system performance were limited by the technology available at that time. With the rapid evolution of high speed, high resolution cameras, high-powered processors, large memory systems, and advanced software, this project extends the concept of real-time insect flight tracking and analysis, revealing patterns of behavior hitherto unobserved and beyond the reach of interpretation. In essence, this research focuses on the development and evaluation of a tool that is considered to have a wide range of applications in the study of insect flight.

## Materials and Methods

The data were collected from two beehives in a single site located at the rooftop of Kilburn Building in Manchester, UK. Videos were recorded across three sessions during June and July 2019, each lasting for approximately 1 h. A Netatmo weather station was installed next to the hive to monitor temperature and rainfall. The weather was sunny and warm (temperature 25 ± 0.5°C at the time of recording) with a light breeze and occasional cloud cover. To explore the influence of windspeed on bee flight behavior, a 2D ultrasonic anemometer was also deployed. Although its sampling frequency was too low to be used to calculate instantaneous air and ground speed vectors, it provided a useful average windspeed every 5 min. Therefore, data collected when the windspeed was close to zero was selected as valid and analyzed on the assumption that the wind had a minimal impact on bee maneuverability or behavior.

**Fig. 1.**
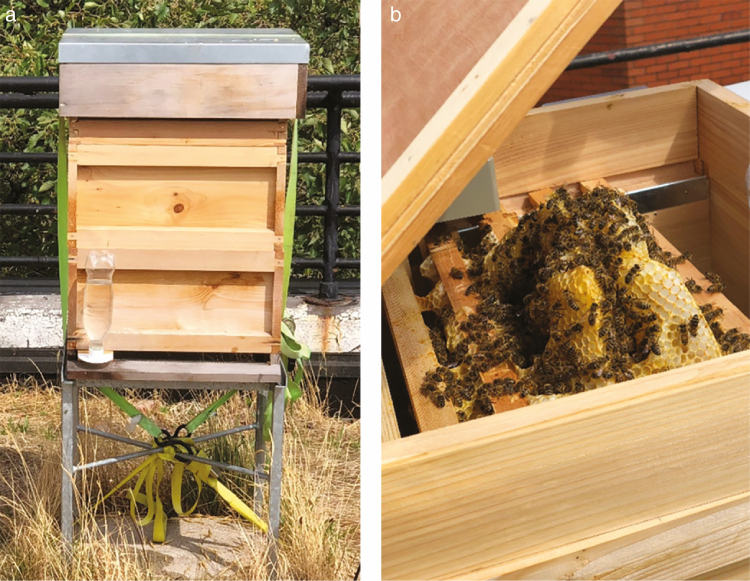
The artificial beehive observed during the experiment (left) and its interior (right).

The two beehives were placed approximately 9 m from each other and were of the same dimension (50 cm × 50 cm × 75 cm with lid on). Figure 1 shows the close-up shot of one beehive and its interior. The beehives were never opened, and all the honey bees were silently observed until the recording terminated. This coincided with the original intention of the observation and one of the most important features of the whole system—to avoid disturbing the bees, which might affect their flight behavior and to extract the honey bee flight behaviors that represent natural activity.

### Hardware and Data Acquisition

**Fig. 2.**
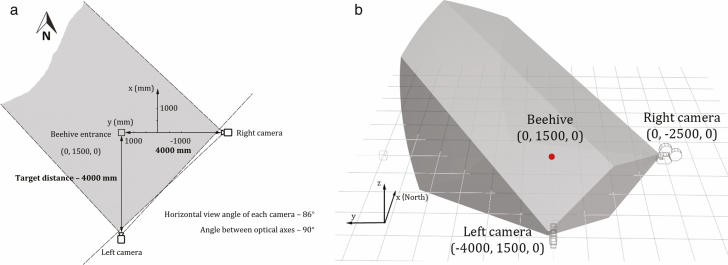
(a) Top view of the system setup, showing the distances between the cameras and the hive, public field of view (gray area), and the location of the hive in world coordinates. (Unit: mm). (b) A rendered 3D shape of the effective observation volume. The red dot marks the location of the beehive in world coordinates. (Unit: mm)

The video acquisition system comprised two GoPro HERO7 cameras, positioned in front of and at the right-hand side of the target (Fig. 2a). Each recorded at a resolution of 2704 × 1520 pixels (2.7K) at 60 frames per second videos, employing a linear lens. Videos from left and right channels were synchronized using a momentary pulse from a mobile phone’s flashlight, observable by both cameras in the overlapping volume (equivalent to a clapperboard strike). To reconstruct offline 3D coordinates and 3D trajectories, the system used an orthogonal configuration, in which the principal axes of the two cameras intersected and formed a 90° angle at the position of the target. The combination of the 2D coordinate pairs was then achieved through appropriate 3D geometry mathematics.

**Fig. 3.**
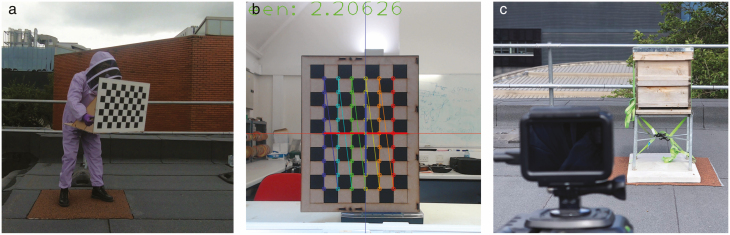
(a) Cameras undergoing calibration using a chessboard pattern. (b) The interface of the calibration software, with alignment lines shown in different colors and the pixel-wise length of the normal vector (z axis) shown at the top.

The calibration of the camera positioning required the use of two 11 × 9 monochromic chessboard patterns of 600 mm × 420 mm dimensions, painted onto two MDF boards and attached to form a 90° angle. The boards were placed next to the target volume before recording, with one facing the front and the other to the right. The alignment commenced with activating the calibration software (Fig. 3b). When the green (depth) axis reached its shortest length (aiming at a length of less than 3 pixels) and the red (horizontal) axis and the blue (vertical) axis were perfectly aligned with the calibration lines marked on the chessboard, it was considered that the visual plane of the camera was parallel to the calibration board and the principal axis coincided with the normal vector coming from the original point on the board. This meant that the two cameras were in an ideal orthogonal configuration. The boards were then removed for the recording phase. To double check the accuracy of the angle and minimize the error, after each recording session, one of the calibration board was raised manually and moved across the overlapping field of view in different orientations, as the reference for further stereo calibration (Fig. 3a). During the experiments, the cameras were positioned 3 m from the calibration board and approximately 4 m from the target beehive. The maximum accepted target distance was set to 10 m away from each camera, beyond which bee contours were excluded by a contour area filter, since the precision of tracking was considered not guaranteed. The resulting effective observation volume was approximately 204 m^3^ (Fig. 2b), which can be easily extended using higher resolution settings. The 35 mm lenses fitted onto the video cameras had an equivalent focal length of 17 mm and a vertical and horizontal field of view of 71° and 86°, respectively. This allowed a sizeable volume to be imaged, providing extensive data on the bees moving in 3D space.

### Analysis and Software

**Fig. 4.**
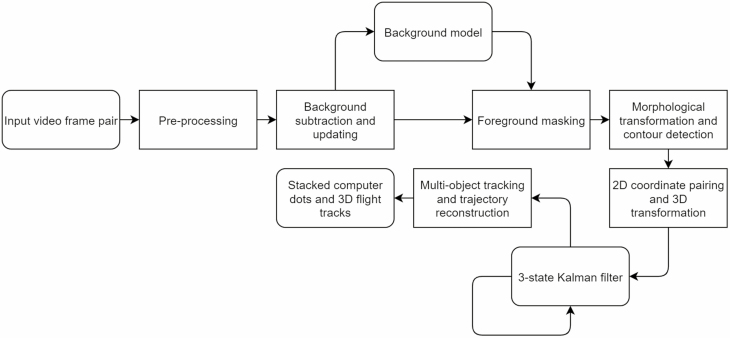
Schematic of the 3D tracking system architecture.

The video analysis software comprised five main processing stages, after which the software generated 3D trajectories for multiple moving targets in the same scene, along with their flight statistics. The structure of the software is shown in Fig. 4. In this section, we describe the methodology used to extract the data from the background, and provide mathematical details on the generation of 3D tracks by combining information from the two cameras. Readers who wish to skip this section may wish to move directly to the Results and discussion.

#### Stage 1: Preprocessing

Data preprocessing was required after obtaining the dual-channel video recordings. This comprised the validation of crucial video elements, such as proper white balance, resolution, the integrity of files, and the compensation for severe vibration or occlusion. In addition, other operations included video format conversion, cropping and synchronization with the flashlight signal. The entire videos were saved as several 5-min, 24-bit uncompressed AVI files.

#### Stage 2: Background subtraction

In this stage, video data from each channel passed through a set of image filters consisting of a background subtractor employing a Mixture of Gaussians (MOG) Model (KaewTraKulPong and Bowden 2002). The MOG subtractor removed the majority of the stationary pixels in the scene and further excluded objects based on the pixel intensity variations caused by their motions, which was controlled by the sensitivity threshold. It also compensated for slight camera shake and detected the shadows and the inclusion of foreground objects rather than merely the outlines. The threshold of defining the ‘stationary state’ could be adjusted in the algorithm used, allowing moving objects to be distinguished from stationary ones. Hence bees could be differentiated from other dimensionally small noise sources, for example the grain generated in low-light conditions using high camera ISO (the sensitivity of the image sensor). The entire background subtraction procedure was integrated with a Gaussian blurring filter to minimize granular background noise, together with a morphological dilation filter (defined in (Haralick et al. 1987)) to enlarge the areas of useful contours and merge neighboring pixels. This stage carried the heaviest computational load for the whole procedure and was the most time consuming. Each 5-min 2.7K 60 frames per second video required approximately 15 min to process, using a laptop featuring an Intel i7-8750H CPU and Nvidia RTX2070-MQ GPU with GPU acceleration enabled during the processing. It is worthy of note that without GPU acceleration, the processing would have required approximately 10 h. The main factors determining computational load were the number of Gaussian models in the subtractor and the number of historical video frames included.

**Fig. 5.**
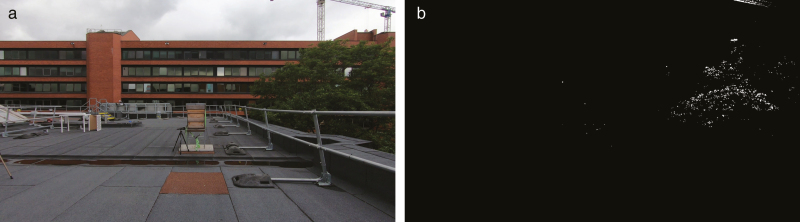
View from the left camera (left) and its binarized image after the processing of background subtraction (right).

The output video frames from this stage were a set of binarized images, shown in Fig. 5. In these images, pixels in the scene are either white (useful data) or black (data ignored). Locating bee positions using the human eye is difficult, but here the algorithm accomplished this with excellent reliability and accuracy. Information redundant to the analysis such as the beehives, buildings, trees, and other objects were almost entirely removed, with any remaining artifacts filtered out in the following stages.

#### Stage 3: Morphological transformation, probabilistic filtering, and contour detection

**Figure 6.**
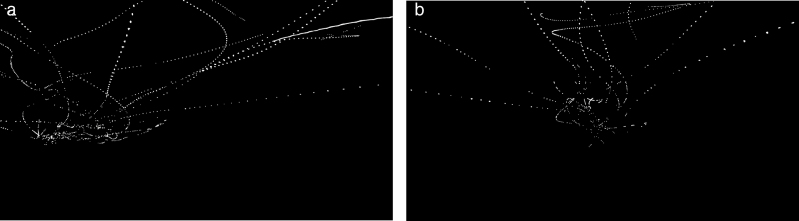
Accumulated computer dots of target locations from left and right camera in a 10-s-long video clip.

Further morphological transformations were applied to both video channels, including an opening operation, which is an erosion followed by a dilation using the same structuring element for both operations (defined in (Haralick et al. 1987)). This removed most of the particle noise and highlighted the desired object contours. The contours of the bees and the remaining noise from objects such as tree leaves and grass were then distinguished according to their probabilities of appearing across the entire scene, since a certain leaf was very likely to remain in a limited space, whereas a bee could appear in almost any location in the scene. The entire binarized video was divided into clips of 10 s duration and a mask image was generated for each clip by accumulating and then normalizing the corresponding pixels values. In these mask images, pixel values of bee locations were very small while those from other objects moving within a smaller space were much higher. Higher values were suppressed with a threshold (Fig. 6) and the mask images were then applied to the original binarized video clips, suppressing noise having no resemblance as bee motion as a result. Image contour division and detection was then implemented, based on the contour area and contour convexity, in which contours with an area of between 10 and 150 pixels and a shape which was rounded rather than strip-like were identified. Thresholds were set to ensure smooth contours and identify bees within the field of view. The centre of mass of every single valid contour, which was considered to be the target location, were computed and saved as the input for the next processing module.

#### Stage 4: 2D coordinate pairing and 3D transformation

Contour information that was stored in two individual channels was now combined. An epipolar coordinate system was constructed, based on: the camera positions during recording, the intrinsic matrices of the cameras and the target distances (the term ‘epipolar’ describes the geometric relationship between two perspective cameras). Then the rotation matrix **R**, the translation vector **T**, the calibration matrix **E,** and the fundamental matrix **F** were defined, referring to the stereo calibration results from the chessboard pattern. More information on the fundamentals of camera models and epipolar geometry can be found in (Hartley and Zisserman 2003)—Part I&II. The epipoles were then computed. Given a 2D point in the left scene, **x**, the epipolar line on the right scene **l’** is given by


$$\begin{array}{l} l^{\prime }=e^{\prime }\times (P^{\prime }P^{+}x)={\left[e^{\prime }\right]}_{\times }P^{\prime }P^{+}x \end{array}$$ (1)

where **e’** is the right epipole, **P** is the left camera, which is a matrix that represents the mapping from the scene to the image and is therefore called a ‘camera’, **P’** is then the ‘right camera’, ${\left[e^{\prime }\right]}_{\times }is\;the\;notation\;ofthe\;skew-symmetric\;matrix\;of\;e^{\prime }$, **P**^***+***^ is the *Moore-Penrose pseudoinverse* (Moore 1920, Penrose 1955) of matrix **P**. Note that the position coordinates × must be converted to homogeneous coordinates before the transformation.

Each epipolar line generated from each target position on the left scene was drawn and labeled on the corresponding right scene. Since the epipolar lines are defined as lines passing through the right epipole **e’** and the corresponding coordinates of the left ones, the shortest distance from every detected point on the right scene to every epipolar line was then calculated, converted and saved into a correspondence matrix. The pairing conditions were defined as being the closest point from the epipolar line and being smaller than the correspondence threshold (in this case 0.003). Points on the right scene that satisfied both conditions were considered as the matched points of the left target locations. The real world coordinates of point P(*x*_*p*_*, y*_*p*_*, z*_*p*_) are given as


$$\begin{array}{l} x^{\prime }=\frac{S\alpha x_{r}\left(\;f+\alpha x_{l}\right)}{f^{2}-\alpha^{2}x_{r}x_{l}}\;and\;y^{\prime }=\frac{S\alpha x_{l}\left(\;f+\alpha x_{r}\right)}{f^{2}-\alpha^{2}x_{r}x_{l}} \end{array}$$ (2)

$$\begin{array}{l} x_{p}=x^{\prime }cos\;\theta -y^{\prime }tan\;\theta \;cos\;\theta \;and\;y_{p}=x^{\prime }sin\;\theta +y^{\prime }\;(\frac{1}{cos\;\theta }-tan\;\theta \;cos\;\theta ) \end{array}$$ (3)

$$\begin{array}{l} z_{p}=\frac{S\alpha y_{l}\left(\;f+\alpha x_{r}\right)}{(f^{2}-\alpha^{2}x_{r}x_{l})\;cos\;\beta_{l}}\;or\;z_{p}=\frac{S\alpha y_{r}\left(\;f+\alpha x_{l}\right)}{(f^{2}-\alpha^{2}x_{r}x_{l})\;cos\;\beta_{r}} \end{array}$$ (4)

where S is the target distance from the calibration chessboard to each camera, α is the inch to pixel scalar, f is the focal length of the camera (17 mm), $\theta \in (-90{}^{\circ },\;90{}^{\circ })$ is the angle between the optical axes of the two cameras, $\beta_{l},\;\beta_{r}\in (-90{}^{\circ },\;90{}^{\circ })$ are the elevation angles of the left and right cameras respectively, and (*x*_l_, *y*_l_), (*x*_r_, *y*_r_) are the 2D coordinates of the target object on left and right view planes respectively.

#### Stage 5: Kalman filtering and Hungarian algorithm

The final part of the software introduced a three-state Kalman filter to smoothen the moving trajectories and to predict the flight pattern of the target. Based on the historical velocity, motion direction and current orientation of existing tracks, as well as the abilities of bees to make sharp turns, accelerate and decelerate, the 3D tracking module distinguished multiple targets during their convergence and kept track of the correct one. The Kalman filtering model was built with three state variables and their derivatives (3D coordinates and 3D velocity) as the state vector. The covariance matrix of the process noise **Q** and the covariance matrix of the observation noise **R** were defined based on a set of simulation results and were adjusted to provide reference values with different weights between historical data and current measurement. The whole group of discrete 3D coordinates in every video frame was fed into the filter to initialize and in return optimize the filter itself. After initialization of the whole model, new detections grabbed from the current frame were allocated to existing tracks through a methodology called the Hungarian Algorithm (Kuhn 1955). It calculated a 2D cost (distance) matrix between each newly detected object and all the existing tracks and output an allocation strategy with a minimum overall cost. This, in the first place, attached every object in the current frame to its nearest existing track based on the cost matrix. Unallocated points, due to their distance from all the existing tracks being too large, were considered as the starting points of new tracks. Existing tracks that obtained no allocation were marked as ‘frame skipped/loss’ and were paid more attention as the filtering process continued, whether their tracked targets had disappeared permanently or merely for a couple of frames. The processing upon the dual channel binarized video yielded a set of complete 3D tracks for a 5-min video. The result was visualized with a color-coded interface using VTK (The Visualization ToolKit).

Analysis based on the curvature of the tracks was conducted. This was done by calculating the change in flight orientations across the whole track, given by


$$\begin{array}{l} \alpha_{i}=cos^{-1}\frac{v_{i}\cdot v_{i+1}}{|v_{i}|\cdot |v_{i+1}|}\;(i=1,\;2,...,\;N-1) \end{array}$$ (5)

$$\begin{array}{l} A=\left[\begin{array}{llll} \alpha_{1} & \;\alpha_{2}\; & \cdots & \;\alpha_{N-1} \end{array}\right] \end{array}$$ (6)

Where *α*_*i*_ is the angle between **v**_*i*_ and **v**_*i*+1_, the instantaneous velocities at measurement point *i* and *i*+1, respectively, N is the length of the track, and **A** is the vector containing the array of α across the entire track. If more than 90% of elements in A are smaller than 5°, the track is marked as a ‘straight track’, otherwise a ‘curved track’.

### System Evaluation and Error Analysis

In order to evaluate the performance of the software, different metrices were defined to reflect the quality characteristics. Let TP stand for the true positives which hold the number of objects correctly labeled as bees and assigned to their own tracks, TN stand for true negatives which hold the number of objects correctly labeled as background, FP stand for false positives which hold the number of non-bee objects labeled as bees or bees that were incorrectly assigned, and FN stand for false negatives that hold the number of bees incorrectly labeled. Then the evaluation metrics are defined as:


$$\begin{array}{l} Recall(Re)=\frac{TP}{TP+FN} \end{array}$$ (7)

$$\begin{array}{l} Precision(Pr)=\frac{TP}{TP+FP} \end{array}$$ (8)

The raw video was processed with a range of background subtraction thresholds/sensitivities (9, 12, 16, 25), morphological transformation kernel sizes (3, 5, 7), probabilistic filtering thresholds (3–10/255) and other filtering parameters, to generate the binarized video. Two 5 s duration image sequences (300 frames each) were randomly selected from each minute of the entire video and were carefully examined frame by frame over different filtering criteria. The examination of bee locations from the raw videos was impossible for human eyes, but that from the binarized video was much easier. The examination focused on the decrease of TP and FP, and unusual patterns (such as abrupt changes in direction, long gaps between adjacent measurement points) in the mask images (Fig. 6). The following parameter setting was used for optimal performance with a high Pr value and a relatively lower Re value, since it was contradictory to maximize both:

- maximum accepted correspondence in Epipolar line searching – 0.01
- background subtractor length of referred historical frames – 400, threshold – 25
- morphological transformation kernel shape – ellipse, kernel size – 5 × 5
- Gaussian filter kernel size = 5 × 5
- probabilistic filtering threshold – 4/255.

Over the examined image sequences, the reduction of TP was lower than 12% on average, i.e., Re was higher than 0.88 within the effective observation volume. The number of frames where FP being non-zero was around 8%, yielding a Pr value of over 0.9.

After Kalman filtering, location outliers were further excluded. The maximum accepted number of skipped frames was set to 6, which terminated tracks that could no longer be detected for 6 sequential frames. Additionally, only tracks with more than 50 measurement points and fewer than 10 missing frames in total were accepted for analysis. Such operations increased Pr further but reduced Re.

The following list defines the main situations when the bees cannot be properly detected, or the 3D pairing fails to work:

1. The target leaves the observation volume of the system.
2. The target inside the volume cannot be observed by one or both the cameras due to occlusion.
3. The target shows very little difference in pixel intensity against the background.
4. The target’s motion slows and no longer triggers the background subtractor.

Most of the above situations can be solved easily using better equipment, e.g., cameras that support higher resolution and recording frame rate, or higher pixel bit depth (10-bit or higher).

The following list describes the main sources of measurement errors:

1. Resolution of the image sensor: with the current resolution setting (2704 × 1520), the length of one pixel on the image sensor represents around 0.48 mm on the plane 3 m away from the camera and around 1.61 mm at 10 m. Such resolution is acceptable considering that the average body length of a Western honey bee is around 15 mm.
2. Bias in the estimation of object centre of mass during the image processing: taking the detection of the head or the tip of the wing of the bee as its location results in a maximum possible error of 7.5 mm.
3. Image distortion from poorly manufactured camera lens: both cameras were calibrated with 50 chessboard images to minimize the effect of lens distortion. The remaining error was further validated during the 3D pairing stage.
4. Imperfect synchronization of the cameras: the maximum temporal offset between the cameras is 1/120 s. Considering the highest flight speed of a honey bee (~7.25 m/s), such offset results in an error of around 60 mm. However, this error can be controlled through the Epipolar constraint during 3D pairing. The relatively strict threshold used in the system was able to detect large synchronization offset and only accepted video pairs with an error smaller than approximately twice the length of the bee (30 mm). And obviously, this type of error can be reduced using a higher frame rate or a wired synchronization approach, which however increases the cost as well as the setup difficulty of the system and reduces the portability.
5. Imperfect measurement of the angle between the cameras: the alignment of the cameras to form a 90° angle was achieved using the software shown in Fig. 3b and the rotation matrix was then optimized using 10 feature points in the scene. The average alignment error over eight trials was ±0.23° (validated through stereo calibration), which was within the Epipolar constraint.

## Results and Discussion

The analysis capabilities of the software are comprehensive; not only can it provide detailed information on the flight statistics for a single bee, it can also generate flight density profiles within the observation volume using the agglomerated track data. Further, it can filter track types according to flight profile, distinguishing between straight trajectories (bees exiting or entering the hive for foraging purposes) and curving tracks, which suggests patrol behavior or orientation flights (Capaldi et al. 2000). Broadly, the software analysis involves three stages of processing:

1. Tagging of bees in the observation volume
2. Extraction of coordinates and binarization
3. Attribution of a set of points to a given track

**Fig. 7.**
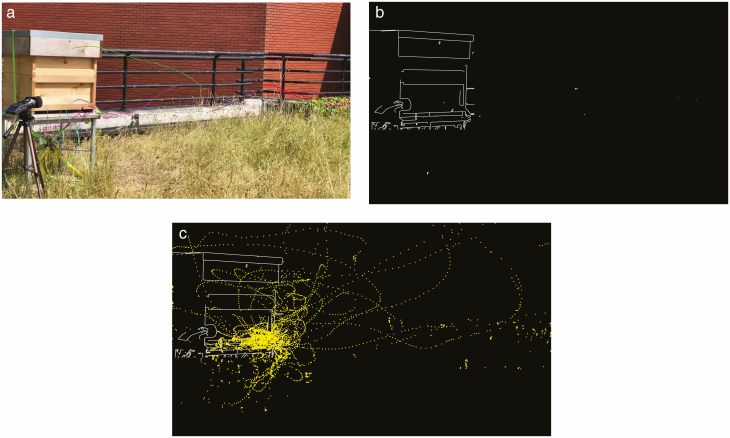
(a) Flight track estimation superimposed with raw video. (b) Binarized detection results after background subtraction. c Accumulated bee locations in historical frames.

**Fig. 8.**
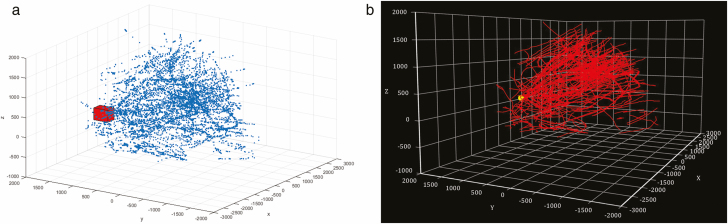
(a) 3D Distribution of all detection points in a continuous 10-min recording. Red cube represents the location of the beehive entrance. (Unit: mm). (b) 3D projection view of the reconstructed flight tracks in a continuous 10-min video, yellow dot marks the location of the beehive entrance. (Unit: mm).

Figure 7 illustrates these stages in the perspective of one of the cameras. Figure 7a shows a freeze-frame from a video, in which multiple bee tracks have been identified and color coded by the software. Figure 7b shows another freeze-frame, in which binarized points have been extracted. And Fig. 7c depicts the accumulated detection points, with all extraneous background removed. Figure 8 illustrates the results of the 3D coordinate pairing and 3D track reconstruction, showing (a) the cloud of all the 3D detection points within the observing volume and (b) the reconstructed collection of 3D tracks after the processing of frame-by-frame Kalman filtering.

### Individual Track Analysis

Since the cameras operated at 60 fps, it was possible to extract information from each track in the public field of view with such a high sampling rate. Table 1 provides an abridged statistical summary for the trajectories of four selected tracks; for the sake of brevity, we have omitted angular velocities, altitude to hive and other secondary features. For each track, the table includes maximum, minimum and average velocity, the maximum, minimum and average accelerations, the instantaneous orientation of and the valid detection points in each track. The orientation vectors are represented as normalized 3D unit vectors and the positive directions of each axis are North, West and vertically upward respectively. It is evident that such data may be used as correlates with a number of other factors, including meteorological conditions, season, general health of the hive population, impact of local environmental conditions and, possibly, stressors including disease and the effects of insecticides. For such correlations to be made, it would require the curation of a sizeable database, involving many hives in different regions, and the acquisition of quantified statistics that defined the above factors. This would be a demanding task, but the use of automated instrumentation—of which this video analysis system is an example—would, over time, facilitate the process.

**Table 1.** Flight statistics from four tracks

	Max. Velocity(m s^-1^)	Orientation(normalized as 3D unit vector)	Min. Velocity	Orientation	Mean Velocity	
Track 01	1.58	(0.08, 0.75, 0.66)	0.60	(0.02, 0.99, −0.15)	0.95	
Track 02	1.84	(−0.40, 0.92, −0.04)	0.83	(0.38, 0.88, −0.30)	1.35	
Track 03	1.95	(−0.23, 0.22, 0.95)	0.24	(0.02, −1.00, 0)	0.75	
Track 04	2.06	(−0.33, 0.86, −0.40)	1.60	(−0.13, 0.86, −0.50)	1.87	
	Max. Acceleration(m s^-2^)	Orientation	Min. Acceleration	Orientation	Mean Acceleration	Track Length
Track 01	21.20	(0.08, 0.49, 0.87)	3.70	(0.36, −0.71, −0.60)	6.20	26
Track 02	22.82	(−0.89, 0.44, −0.14)	3.17	(−0.74, −0.16, 0.66)	9.00	46
Track 03	30.78	(−0.94, 0.33, 0.11)	7.75	(0.91, 0.29, 0.30)	8.25	99
Track 04	15.10	(−0.76, −0.62, −0.22)	3.99	(−0.04, 1.00, 0.05)	7.34	23

### Aggregated Data Analysis

**Fig. 9.**
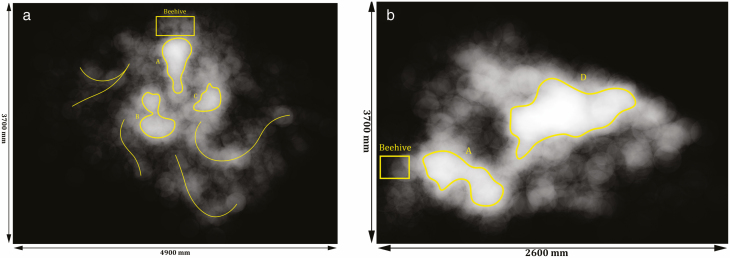
Density map of flying honey bees around the hive showing all tracks from (a) the top view and (b) the side view. The location of the beehive entrance is marked with a yellow rectangle. Areas that are marked by closed yellow boundaries (A, B, C in (a) and A, D in (b)) were more frequently visited by the worker bees than the rest of the area. Similarly, the yellow curves are the tracks that were more frequently taken based on the thresholding of pixel intensity.

In addition to the generation of the 3D distribution of all the detection points (Fig. 8), Fig. 9a shows the monochromic heatmaps of the density of honey bees in the observation space. Since the size of a measurement point (1 mm^3^) is much smaller than the dimension of the observation volume, each point in Fig. 7 was expanded to cover a larger area, which blurs the density map but reveals hitherto hidden patterns of distribution. Darker areas were less visited while brighter areas were more populated over time. These heatmaps were then processed with a selected pixel intensity threshold to show areas that were most populated, namely population clusters. Figure 9a shows the highly populated cluster not only at the entrance (cluster A) but also approximately 1.5–2 m away from the entrance (cluster B and C). Cluster B and C located at both sides on the way to the entrance. Figure 9b shows the heatmap of honey bee density from a side view. It can be seen that cluster A (the same cluster as that in Fig. 9a) is lower than cluster D, and cluster D is most likely the superposition of cluster B and C, since they are located at the same height. The connection between cluster A and D is very narrow, which possibly means bees tended to choose the same path when flying across different clusters. Further, the location of cluster D is not on the main route to the hive, instead it suggests the hovering of the patrolling bees. These clusters may relate to bees’ switch of senses at close proximity to the hive indicated by (Butler 1949).

Fig. 9a also shows several flight tracks that are more frequently taken—‘popular tracks’. From previous studies on honey bee foraging behavior, it was found that when a bee discovers a profitable food source and starts to carry food back to the hive, the foraging target does not change until abandoned (Karaboga 2005). The bee will take several round trips to the same food source and also share the information through waggle dances to other bees. This means a stable food carrying chain is established. Such a bee is often referred to as an ‘employed’ bee. The ‘popular tracks’ shown in Fig. 9a are probably the tracks of employed bees and also indicate the direction of the food source.

**Fig. 10.**
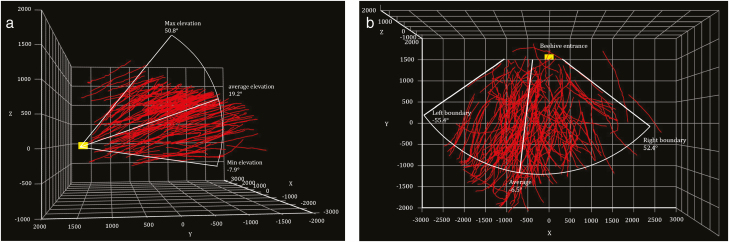
Low curvature tracks—‘straight tracks’—shown inside view (left) and top view (right). Yellow rectangle represents the location of the hive entrance. (Unit: mm).

From the analysis based on the curvature of the tracks, there are altogether 204 ‘straight tracks’ out of 605 tracks, shown in Fig. 10. Out of 204 tracks, only 12 have a negative elevation angle from the entrance, which means it is very rare for a bee to fly lower than the hive entrance in a straight path and the straight tracks are almost exclusive to bees’ leaving or returning behaviors (the hive was positioned on a metal frame at a height of approximately 600 mm from ground). The elevation angle ranged from −7.9° to 50.8°, with an average of 19.2° as shown in Fig. 10, left. When observed from the top, the angle of the sector covering the tracks was from −55.4° to 52.4° (assuming north as positive direction), with an average of −6.5°. Out of the 204 straight tracks analyzed during the observation, 97 were marked as ‘exiting tracks’ and the rest 107 were ‘entering tracks’. The difference was not significant, implying that many foragers made their way back without undue effort. This supports the conclusion made in another study, showing that experienced foragers take less effort returning, flying into the hive without hesitation (Karaboga 2005).

**Fig. 11.**
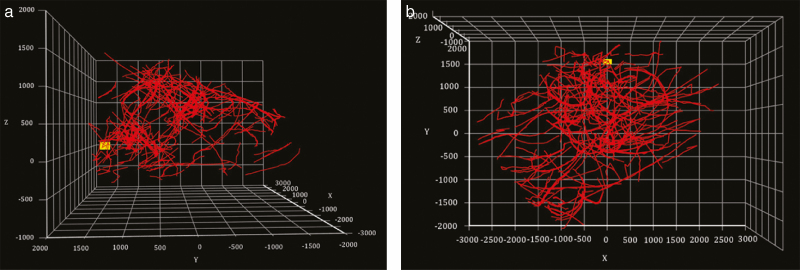
Side view (left) and top view of the tracks with large curvature—‘curved tracks’. (Unit: mm).

Figure 11 shows the side and top view of the remaining tracks, i.e., the ‘curved tracks’. It is interesting to see that the curved tracks seem to have no correlation with the location of the beehive entrance, while most straight tracks either started or terminated at the entrance.

Table 3 provides the statistical summary of the entire bee swarm in the recorded video. It includes several metrics that describe the activity level of leaving and returning workers, which are defined as follows:

The accumulated magnitude of velocities of all historical locations during the flights – *I*,


$$\begin{array}{l} I=\sum_{n=1}^{N}\sum_{l=1}^{L_{n}}v(n,l) \end{array}$$ (9)

The ratio between the average velocities of entering and exiting flights – *r*,


$$\begin{array}{l} r_{exit-to-enter}=\frac{I_{exit}/N_{exit}}{I_{enter}/N_{enter}}, \end{array}$$ (10)

Where *v*(*n, l*) stands for the magnitude of the instantaneous velocity on the *l*-th location of track *n*, *L*_*n*_ stands for the length of track *n*, in terms of the number of historical locations, *N*_*enter*_*and N*_*exit*_ stand for the number of tracks of entering and exiting bees, respectively, in a 1 min long video, and *I* is calculated individually for entering and exiting tracks as *I*_*enter*_*and I*_*exit*_. The table provides detailed information on how the activity level of the bee swarm changed as time passed, based on *N*_*enter*_*and N*_*exit*_. *r*_*exit–to–enter*_ also shows that the average speed of exiting tracks was slight higher than that of entering tracks, but not by a large factor, possibly due to their food load, depletion of stamina, or frequent navigation updates that drag.

## Online Video Data

Much of the video data generated by this research is now available online for public use. Links to some of the videos that relate to the above analysis are given in Table 2. These videos demonstrate the intermediate results from one camera and the final 3D visualization. They were either exported from the software described above or screen recorded by the author.

**Table 2.** Links to videos illustrating stages in flight trajectory extraction

Description	Link
Binarized bee locations after background subtraction.	https://drive.google.com/file/d/14g8zdUL6p4SNlyfkPQy04cVb0_CRJCd2/view?usp=sharing
Historical locations of all the detected bees that is continuously updated.	https://drive.google.com/file/d/1hmem5bIeXDiSDZzNVza5Ecc4cQ0g_LlB/view?usp=sharing
Collection of the finalized historical locations shown in 3D view.	https://drive.google.com/file/d/1Iz-hQi7V9opVVWm0dzR6pHHPse-c9qT3/view?usp=sharing
Rotational view of all reconstructed 3D flight tracks in a dual-channel recording.	https://drive.google.com/file/d/1YwullTGd0PQ9GXPex16aM2HgFMMq6k0n/view?usp=sharing

**Table 3.** Flight statistics of the entire bee swarm in the recorded video

Time segment(min)	*N*_*enter*_	*I*_*enter*_ (m/s)	*N*_*exit*_	*I*_*exit*_ (m/s)	${}^{I_{enter}}╱{}_{N_{enter}}\;$	${}^{I_{exit}}╱{}_{N_{exit}}\;$	*r*_*exit–to–enter*_
0–1	68	3,934.3	70	4,180.58	57.8584	59.7226	1.0322
1–2	28	1,703.8	40	2,463.00	60.8521	61.5750	1.0119
2–3	26	1,425.2	19	1,253.25	54.8173	65.9605	1.2033
3–4	46	2,370.7	30	1,678.23	51.5385	55.9410	1.0854
…	…	…	…	…	…	…	…
8–9	22	1,105.9	11	653.40	50.2718	59.4004	1.1816
9–10	17	856.45	15	850.74	50.3795	56.7161	1.1258
Sum	340	1,8891.5	265	1,5771.0	-	-	-
Mean	-	-	-	-	55.5633	59.5147	1.0711

## Conclusion

Prior to this research, instrumentation and monitoring systems used for other studies were neither appropriate nor optimized for the study of bee behavior, especially flight behavior. Despite the recent advancement in real-time digital video image processing, their application in the analysis of insect behavior has not been widely introduced. The system described in this paper was designed to work in the field and did not interfere with normal bee flight or activity. It therefore proved the feasibility of applying classical image processing techniques to the study of insect motion. It was found that:

- the returning workers have a smaller average velocity than the departing ones, possibly due to the carrying load and stamina depletion during foraging.
- compared with departing from the hive, returning workers normally do not need much navigation of the entrance to the hive when they are near the hive. Instead, the margin between the number of bees leaving and entering the hive directly was very small. And this ability can possibly be trained as the worker gets more experienced.
- the workers hovering in front of the hive entrance tend to form small clusters that are slightly higher than the entrance. Additionally, bees flying from one cluster to another often move along a more popular track, forming what is named as a ‘flight tunnel’.

The 3D bee tracking system was capable of extracting the 3D coordinates of most detected honey bees. Further analysis based on such measurement of bees not only shows the flight statistics such as velocity and acceleration, but also provides a clearer view of the inflight behavior of a bee at any point along its track. Although the flight of one individual may merely be an occasional behavior of which the features are not representative at first sight, the analysis of the entire swarm provides a strong supportive evidence of conclusions and interpretations from other studies and the data volume is much larger in this research. The software was capable of uniquely extracting the flight trajectories of individual bees, obviating the requirement for physical marking using colored dots or tracking tags, which inevitably influences behavior. A wide range of behaviors within the detection range was recorded and analyzed with unprecedented detail and precision in comparison to other studies. Continued use of the system may in time lead to the discovery of as-yet undiscovered phenomena.
